# Genetic Implications of Male-Reproductive-Health-Associated Comorbidities

**DOI:** 10.5152/tud.2022.20463

**Published:** 2020-10-30

**Authors:** Nahid Punjani, Caroline Kang, Dolores J. Lamb

**Affiliations:** 1 James Buchanan Brady Foundation Institute of Urology, Weill Cornell Medical College, New York, NY, USA; 2 Englander Institute for Precision Medicine, Weill Cornell Medical College, New York, NY, USA; 3 Center for Reproductive Genomics, Weill Cornell Medical College, New York, NY, USA

**Keywords:** Chromosomal abnormalities, genetic conditions, male infertility

## Abstract

Male infertility is a common problem. There is growing evidence that infertile men may harbor significant illness and disease. Many of the genetic causes of male fertility have implications on other systemic illnesses. This review aims to discuss various genetic conditions and gene mutations and alterations associated with male infertility and evidence for associated systemic conditions. These findings highlight the importance of a thorough workup in men presenting for a fertility assessment.

**Main Points**

- Infertile men may harbor genetic diseases with associated systemic implications.
- Various genetic conditions have been implicated in male infertility including chromosomal conditions, non-chromosomal conditions, disorders of sexual differentiation, and birth defects.
- Men with infertility warrant a thorough work-up and evaluation during their initial presentation.

## Introduction

Infertility is a common problem, affecting up to 15% of couples, with male factor present in up to 50% of these cases.^1^ The exact etiology often remains unclear, which has sprouted research to understand the breadth of disease, which impacts infertility as well as other systemic and genetic diseases.^2^ Although the exact etiology of infertility is unknown for approximately 40% of men, a European study found that up to 25% of men with azoospermia and severe oligozoospermia had genetic abnormalities.^2-8^

In the 1990s, researchers discovered only over 1% of patients assessed at 2 high-volume male infertility clinics harbored significant pathologies ranging from embryologic and endocrine abnormalities to malignancy as a result of genetic and chromosomal anomalies.^3-6^ More recent data suggest that this number is greater, with up to 6% of men being assessed for infertility with underlying chromosomal anoma-lies.^7^

Overall, this information highlights the importance of completing a full assessment of men presenting with infertility, in addition to a standard focused history, physical examination, and semen analysis. Our review highlights the genetic diseases potentially harbored by men presenting with infertility and the associated comorbidities and health implications of these conditions.

## Genetic Conditions

Although the exact etiology of infertility is unknown for approximately 40% of men, a European study found that up to 25% of men with azoospermia and severe oligozoospermia had genetic abnormalities, including cystic fibrosis transmembrane conductance regulator (CFTR) gene mutations, Y chromosome microdeletions, and chromosomal abnormalities.^2,8^ Approximately 1000 genes were identified that could have a direct impact on spermatogenesis as well as associations with genitourinary birth defects and disorders of sexual differentiation, which collectively might contribute to fertility issues later in life.^9-15^ In some instances, genes might be deleted or the copy number of the gene might be increased or decreased (resulting from structural chromosomal anomalies owing to microduplications or microdeletions), conferring a wide range of phenotypes, or there could be epigenetic modifications of the gene, which could modify the expression levels without a structural change in the gene itself.^16^

## Chromosomal Conditions

### Y Chromosome Anomalies

The Y chromosome is an acrocentric chromosome, which has both a short arm (Yq) and a long arm (Yp) separated by a centromere.^17^ Alterations in the genes on both Yq and Yp are implicated in infertility. Numerous genes on Yq affect spermatogenesis (*PCDH11Y, TSPY*, and *ZFY*), and the sex-determining region gene (*SRY*), also located on Yq, encodes the transcription factor necessary for testis development.^18-20^ *TSPY* is thought to function as proto-oncogene and may be associated with development of gonadoblastoma.^21^ *SRY* deletion can result in 46,XY individuals with a female phenotype, termed Swyer syndrome.^22^ These individuals show complete gonadal dysgenesis and are at risk for developing germ-cell neoplasia and, thus, are recommended to undergo immediate gonadectomy at the time of diagnosis.^22,23^

### Y Chromosome Microdeletions

There are 3 azoospermia factor (AZF) loci on the long arm of the Y chromosome (AZFa, AZFb, and AZFc), which encode multiple genes involved in spermatogenesis.^19,20^ Deletions within these loci are known as Y chromosome microdeletions, as these deletions are not identifiable with standard karyotype and require more detailed molecular techniques for diagnosis. It is estimated that Y chromosome microdeletions account for up to 12% of men with non-obstructive azoospermia.^24^ Genes that encode proteins involved in spermatogenesis include *DBY, USP9Y, HSFY, KDM5D, PRY, RPS4Y2, BPY2, CDY, GOLGA2LY*, and *TTY4.*^19^ Men with Y chromosome microdeletions require surgical sperm retrieval using assisted reproductive technology (ART) in order to father offspring. Knowing the location of the Y chromosome microdeletion is critical as the rates of sperm retrieval vary dramatically. Men with complete AZFa and AZFb microdeletions have no reports of successful sperm retrieval; however, in the hands of a highly skilled surgeon, rates of sperm retrieval in men with AZFc approach 50%-60%.^24^

Microdeletions of Y chromosome genes have implications beyond infertility and include other systemic diseases and conditions, such as cardiovascular disease, cerebrovascular disease, neurologic conditions, malignancy (bladder, prostate, and liver), changes in crown tooth size and stature, and genitourinary birth defects (Table 1).^12,21,25-34^

The Y chromosome contains 2 pseudoautosomal regions (PARs) at the tip of each arm, with PAR1 at the end of Yq and PAR2 at the end of Yp.^34^ The PARs are homologous and undergo recombination with X chromosome PARs during meiosis, which is thought to be important for the appropriate segregation of sex chromosomes.^19^ These PARs contain numerous genes, with 16 located in PAR1 and 5 genes located in PAR2.^25^ Mutations of the short stature homeobox (*SHOX*) gene in PAR1 are associated with a musculoskeletal and stature-related phenotypic spectrum of disorders, including Leri-Weill dyschondrosteosis, Madelung deformity of the wrists, bowed wrists, and non-specific short stature, and show a coexisting genomic syndrome present in approximately one-quarter of men with Y chromosome microdeletions.^33^ Duplication of the *SHOX* gene is responsible for the variable height seen in patients with Klinefelter syndrome (KS), whereas homozygous mutations may cause Langer mesomelic dwarfism.^33^ On PAR2, duplication of *VAMP7* significantly affects the rates of cryptorchidism, resulting in spermatogenic dysfunction and is also implicated in external male genitalia abnormalities, such as reduced penile length and hypospadias.^12^

### Structural Y Chromosomal Changes

In addition to the previously mentioned issues, structural changes may occur owing to chromosomal translocation or chromatid fusion after chromosomal breaks, which result in isodicentric Y chromosomes (2 centromeres).^35^ These structural changes may cause gene duplications (from genes on the short arm, such as PAR1 genes) or deletions (from genes on the long arm, such as PAR2 genes) depending on the regions involved and may therefore result in variable phenotypes and mosaicism. Phenotypes may include short stature secondary to *SHOX* gene deletion (up to 80%), ambiguous genitalia (up to 75%), spermatogenic failure, growth delay, language delay, dysmorphic features, autism, mental disorders, and learning difficulties, many of which may be owing to loss of PAR2 genes.^19,36^

### X Chromosome Anomalies

Although the Y chromosome contains genetic material for male development, there are multiple genes on the X chromosome involved with male infertility, the most significant of which result in abnormalities and conditions related to the androgen receptor (AR) gene.

In addition to the AR gene, various X chromosome related genes have also been implicated in male infertility (*TEX11, MAGEB4, RHOX, HAUS7*, and *TAF7L*). The testis-expressed gene 11 (*TEX11)* is a well-described X-linked gene involved in male infertility, which is located at the q13.2 locus.^37^ Since the gene is essential for meiotic recombination, gene mutations of *TEX11* result in meiotic arrest and azoospermia.^38^ Melanoma-associated antigen B4 (*MAGEB4*) is involved in germ-cell differentiation and has been implicated in non-obstructive azoospermia.^39^ Reproductive homeobox on the X chromosome (*RHOX*) genes are expressed in Sertoli cells, and certain gene variants of this family can be present in men with severe oligozoospermia.^40,41^ The TATA-box binding protein associated factor 7 like (*TAF7L*) gene encodes a transcription factor, which shows testis-specific expression and mutations that are associated with spermatogenic failure.^40^ Mutations of HAUS augmin-like complex subunit 7 (*HAUS7*), which is involved in centrosome regulation and cytokinesis, has been described in cases of severe oligozoospermia.^40^

### Kennedy Disease

Kennedy disease, also known as spinal and bulbar muscle atrophy, is a rare and usually adult-onset neurodegenerative condition associated with CAG trinucleotide repeat expansion (>35 repeats) within the AR gene.^42,43^ Because this is a motor neuron disease resulting from diminished transcriptional activation activity of the AR gene in addition to muscular atrophy, Kennedy disease results in gynecomastia, testicular atrophy, and spermatogenic failure depending on the length of triplet repeat expansion.^44^ The age of onset and phenotypic severity of the disease is directly proportional to the length of the full penetrance trinucleotide expansions. Clinically, individuals with Kennedy disease develop progressive oligozoospermia or azoospermia and sexual dysfunction.^45^ At present, conflicting data exists with regard to triplet repeat lengths <35, but these individuals generally have milder symptoms.^46-48^

### Klinefelter Syndrome

Klinefelter syndrome is a genetic condition that includes 1 or more extra X chromosome(s) and is the most common numerical chromosomal abnormality in men. The prevalence is 1/500 of live male births and is believed to occur secondary to chromosomal non-disjunction during meiosis.^2,49^ Klinefelter syndrome is frequently implicated in infertile men (up to 12%) with nonobstructive azoospermia, and the etiology for infertility stems from small testes, low testosterone levels, and fibrosis of the seminiferous tubules.^49^ In the majority (90%) of cases, there is a single extra copy of an X chromosome resulting in a 47,XXY karyotype, but other genotypes may be present, including more than 1 extra copy of the X chromosome (i.e., 48,XXXY or 49,XXXXY), mosaicism (46,XY/47,XXY), or partial supernumerary chromosomal pieces (47,iXq,Y).^49,50^

These men have characteristic phenotypic features, including tall stature; reduced testis size; reduced body, chest, and facial hair; gynecomastia; varicosities of the lower extremities; eunuchoid skeletons; wide hips; narrow shoulders; and absence of frontal balding.^49^ Men with KS have reduced normal testicular tissue secondary to fibrosis and hyalinization of the seminiferous tubules, which begins early in life during the fetal stage and rapidly progresses throughout puberty.^51,52^ There are various hypotheses that explain these changes, including insufficient supernumerary X chromosome inactivation, Leydig cell insufficiency, and deregulation of Leydig and Sertoli cells.^52-54^

Systemic conditions in individuals with KS may include altered intellect, osteoporosis, increased risk of breast malignancy, sexual dysfunction, and low testosterone levels requiring exogenous hormone replacement therapy.^51,52^

### 47,XYY Male

A very rare occurrence involving chromosomal non-disjunction is the 47,XYY karyotype. This condition occurs in 1/1000 of live births and is associated with limited phenotypic abnormalities but may include variability in the testicle size and development (from normal size to atrophic), elevated body-mass index, greater stature secondary to *SHOX* gene duplication, increased risk of learning disability, language issues, and behavioral issues.^2,55-57^ From a fertility standpoint, these men may have hormonal disturbances and variability in sperm quality, including reduced sperm concentration, increased prevalence of hyperhaploid (increased number of unpaired chromosomes) sperm that may transmit an extra chromosome to their offspring, risk of spermatogenic failure with maturation arrest, and Sertoli-cell only histopathologies.^2,55^

### 46,XX Male

This rare condition, also known as de la Chapelle syndrome, occurs in 1/20 000 of live births secondary to *SRY* translocation to the X chromosome or an X chromosomal abnormality in the region responsible for inhibition of autosomal testis-determining genes.^2,58^ Phenotypically, these men may have genitourinary anomalies, including micropenis, persistent Müllerian remnants, hypospadias, and cryptorchidism.^59^ Hormonally, these men tend to have hypertrophic hypogonadism and azoospermia because of the absence of azoospermia factors.^58^

### Kallmann Syndrome

Kallmann syndrome occurs in up to 1 in 10 000 of live births.^2^ Common characteristics include hypogonadotropic hypogonadism and anosmia and less commonly obesity, ocular abnormalities (congenital ptosis and abnormal eye movements), hearing impairment, involuntary limb movements, cleft palate or lip, dental disorders, upper urinary tract anomalies (renal agenesis), and corpus callosum agenesis.^60^ *KAL-1*, a gene encoding a neural cell adhesion molecule, encodes the protein that is most commonly involved in normal hypothalamic development.^61^ Other genes associated with Kallmann syndrome, include *FGFR1, CHD7, WDR11, PROKR2, PROK2, and FGF8*.^57^ Because the main driver for infertility in men with Kallmann syndrome is hypogonadotropic hypogonadism, exogenous treatments, such as testosterone as needed for virilization and gonadotropins for fertility, may be used.^62^

### Other Chromosomal Abnormalities

The incidence of chromosomal translocations in infertile men is 9-fold greater than that in the general population and includes variable degrees of translocations, which may be balanced or unbalanced; some individuals may have some degree of mosaicism.^63^ More specifically, Robertsonian translocations, which occur between the acrocentric chromosomes (13, 14, 15, 21 and 22), have a well-documented impact on male infertility.^64^ These occur in 1/1000 of births and, as with any chromosomal translocation, may have variable balancing and complexity.^64^ The most common translocations include 13q14q and 14q21q. Men with these translocations tend to be phenotypically normal but may present with reproductive difficulty. These translocations may lead to oligozoospermia, monosomy or trisomy in offspring, and spontaneous miscarriage.^65^

In general, infertile men also have an 8-fold higher rate of aneuploidy and chromosomal inversion than fertile men.^2^ When examining a subset of infertile men, 4.6% of men with oligozoospermia and 13.7% of men with non-obstructive azoospermia had chromosomal inversions or translocations.^66^

## Non-structural Chromosomal Conditions That Underlie Asthenozoospermia, Asthenoteratozoospermia, and Teratozoospermia

### Primary Ciliary Dyskinesia

Primary ciliary dyskinesia (PCD) is an autosomal recessive condition that results in male infertility, and patients with PCD also have dextrocardia and chronic rhinosinusitis with an increased risk of bronchial sepsis.^67,68^ As cilia line the respiratory tract, abnormalities result in reduced mucociliary clearance of the airways, predisposing the individuals with PCD to chronic airway infections. Functional ciliary structures also are critical for sperm flagellar tail function; therefore, men with PCD exhibit severe deficits of sperm motility as well as other structural flagellar defects (missing dynein arms, lack of radial spokes, and microtubular translocations).^67,68^ Multiple autosomal genes may be implicated in PCD, including *CCD39, DNAAF1-3, DNAH5, DNAI1/2, DYX1C1, HEATR2, HYDIN, LRRC6, RSPH1, RSPH4A, RSPH9*, and *ZMYND10*, which portends a wide spectrum of possible motility phenotypes as described earlier.^68,69^ Today, over 991 different gene defects are known (>90 validated for clinical diagnostics) affecting the structures of the axoneme, inner and outer dynein arms and their regulatory complex, central microtubule pair, Nexin links, and laterality.

### Multiple Morphological Abnormalities of Sperm Flagella

Multiple morphological abnormalities of sperm flagella (MMAF) is a syndrome associated with male infertility, which includes a spectrum of morphological sperm flagellar abnormalities, including dysplasia of the fibrous sheath or absent or dysmorphic (short, bent, irregular, or coiled) flagella.^70,71^ The principal piece of the flagellum is usually affected, which results in flagellar abnormalities and ultrastructural defects.^70^ Because a normal axonemal structure occurs in a 9+2 format, these individuals usually possess a 9+0 structure owing to the absence of central microtubular pairs.^71^ Potential genes mutated in MMAF include *DNAH1* (better pregnancy rates after intracytoplasmic sperm injection), *CFAP43*,and *CFAP44*, which are responsible for the majority (up to 70%) of cases.^71,72^ Less commonly, mutations of *AKAP4, CCDC39, CFAP69, ARMC2, ORICH2, AK7, CFAP251, CFAP65,CEP135, FSIP2, SPEF2*, and *DNAH2* may be involved.^71,73^ In addition to morphological abnormalities, these patients are at risk of gonosomal disomies and diploidies.^74^

### Aurora Kinase C Deficiency

Aurora kinase C *(AURKC)* encodes a serine/threonine protein kinase that is involved in regulating chromosome segregation during mitosis and is highly expressed in the testis.^75,76^ Men with *AURKC* defects present with primary infertility characterized by teratozoospermia, where the spermatozoa are aneuploid with significantly larger heads (macrocephalic spermatozoa) and additional flagella.^75-77^ The most common *AURKC* defect is a mutation, c.144delC, which results in premature translation termination forming a truncated protein without the kinase domain.^75-77^ Studies of North African infertile men have demonstrated a high carrier rate of the *AURKC* c.144delC mutation with an allelic frequency of 2.14%.^75^

### Young’s Syndrome

Young’s syndrome is a rare condition characterized by male infertility, chronic rhinosinusitis, and bronchiectasis.^78^ Male infertility is affected by spermatogenic dysfunction resulting from axonemal abnormalities and epididymal obstruction that results in azoospermia.^79^

### Globozoospermia

Globe-shaped, round sperm heads, or globozoospermia, is an abnormal sperm morphology in which the heads lack or have atrophied or misplaced acrosomes necessary for egg fertilization.^80^ Various genes have been studied as the etiologic factors for globozoospermia, including *SPATA16, PICK1*,and *DPY19L*,^81^ and *DYP19L2* and *SPATA16* have been clearly demonstrated to be causative.^82,83^ Patients with globozoospermia rely on ART and intracytoplasmic sperm injection (ICSI), but the rates of fertilization and live births are low despite oocyte activation with ICSI and *in vitro* fertilization (ICSI-IVF), and many embryos are at increased risks for aneuploidy.^81,84^

### Cation Channels of Sperm

Mutations of cation channels of sperm (CATSPER) are a known cause of male infertility. These are among many other known ion channels implicated in male infertility, such as the proton voltage-gated ion channel (Hv1), potassium voltage-gated ion channel (SLO3/KCNU1), and sodium voltage-gated ion channel (NaV1.1-1.9).^85^ Of the 4 genes identified in the CATSPER family, 2 are responsible for the infertility phenotype (*CATPSER1* and *CATSPER2)*.^86^ Although both may cause infertility albeit with differential effects on semen quality, *CATSPER1* is non-syndromic, whereas *CATSPER2* is syndromic (deafness-infertility syndrome).^87^ At a semen analysis level, men with *CATSPER1* mutations have oligozoospermia, reduced semen volume, minor changes to sperm motility, and some effect on morphology, whereas men with *CATSPER2* mutations have oligozoospermia, asthenospermia, teratazoospermia, and reduced viability.^87^

## Disorders of Sexual Differentiation

### Androgen Insensitivity Syndrome

Androgen insensitivity syndrome (AIS) is a rare condition that occurs secondary to damaging mutations of the AR gene.^88^ These individuals generally have a 46,XY karyotype and may present with a spectrum of diseases, including partial, mild, or complete androgen insensitivity.^88^ Given this spectrum of AR insensitivity, individuals have varying degrees of virilization and altered external genitalia, including micropenis, undescended testis, gynecomastia, and hypospadias.^88,89^ In complete AIS, individuals present with complete feminization of the external genitalia (but functional cryptorchid testes), whereas those with mild disease may present as undervirilized males.^90^ Those with partial AIS, depending on the regions where the AR is involved, variable expressivity and/or other modifying factors may present with a much wider spectrum of phenotypes that can vary between siblings because of the presence of other biological modifiers.^90^ Other than virilization changes, patients with AIS may be tall, have endocrinopathies, and may present with inguinal hernias.^14^ In some instances, these patients may present only with infertility and may have impaired spermatogenesis and sexual dysfunction.^91^

### Gonadal Dysgenesis

Gonadal dysgenesis is a family of conditions with impaired gonadal development, which ranges from partial to complete gonadal dysgenesis. Various gene mutations are responsible for different types of dysgenesis, including *SRY, SOX9, WT1, SF1, DMRT1, DHH, FO2, NR5A1, GATA4, MAP3K*, and *BMP1*.^92^ More specifically, mixed gonadal dysgenesis occurs secondary to rearrangement or chromosomal missegregation and often results in a 45XO/46XY mosaicism, with up to one-third of patients having a normal karyotype.^93^ These patients present as phenotypically normal males, but some individuals may have some degree of ambiguous genitalia.^94^ Internally, they tend to have a single abnormal testis, often devoid of germ cells, and a contralateral streak gonad. Systemically, these individuals have other associated conditions including cardio-renal malformations and malignancy (germ-cell tumors and gonadal blastomas).^95^

### Five Alpha Reductase Deficiency

Five alpha reductase (5AR) converts testosterone to dihydrotestosterone (DHT), and alteration of 5AR can result in complete or partial enzyme deficiencies.^96^ The AR in the testis relies on testosterone for spermatogenesis, but DHT is required for a ccessory sex organ development. Therefore, individuals with 5AR deficiency have a 46,XY karyotype and normal internal structures, including testicular gonads and Wolffian duct structures (seminal vesicles, vas deferens, epididymis, and ejaculatory ducts), but are phenotypically female owing to the lack of DHT.^96^ Because the appearance of the external genitalia is that of a female, these individuals are typically raised as females; however, during puberty, testosterone surges promote testicular descent, penile growth, and development of a male body habitus.^96^ However, in the absence of DHT, there is limited phallic development.^96^ In addition to reduced phallic length, there may be accompanying hypospadias, which may impair natural conception.^13^ Interestingly, these individuals also have low-volume and viscous ejaculates secondary to poor prostate development from reduced DHT levels and an absence of serine proteases necessary for liquefaction.^13^

### Congenital Adrenal Hyperplasia

This autosomal recessive condition occurs secondary to various enzyme defects in the normal steroidogenesis pathway. The most common of these includes 21-hydroxylase deficiency.^97^ The steroidal pathway is responsible for the production of glucocorticoids, mineralocorticoids, and androgens; depending on the enzymatic defect, various deficiencies and/or combinations may occur. In patients with congenital adrenal hyperplasia, fertility ranges from 23% to 67%, which may be secondary to intratesticular adrenal rest tumors, which may cause gonadal damage or hypogonadotropic hypogonadism from negative feedback of excess androgens produced from the adrenal gland.^97^ Patients with congenital adrenal hyperplasia are also at an increased risk of developing adrenal tumors and hyperplasia, short stature, insulin resistance, and cardiovascular disease.^98^

### Persistent Müllerian Duct Syndrome

This disorder is characterized by the persistence of structures formed by the Müllerian duct, including the uterus, cervix, fallopian tubes, and upper two-thirds of the vagina.^99^ This phenotype develops secondary to gene mutations of either anti-Müllerian hormone (*AMH*) or its receptor (*AMHR2*).^100^ These individuals have a 46,XY karyotype and are at an increased risk for cryptorchidism or testicular ectopia and subsequently have an increased risk of certain malignancies, such as teratomas, yolk sac tumors, and embryonal tumors.^99^ Although fertility is limited in these individuals and they have azoospermia, rare cases have been reported in those with a scrotal testis and associated vas deferens and epididymis.^100,101^ These individuals may also develop obstructive causes of infertility secondary to iatrogenic injury, which may occur during the removal of persistent Müllerian remnants.^102^

### 1Birth Defects

There has been emerging evidence that male infertility is linked to genitourinary birth defects. Individuals with these defects may also be harboring additional systemic disease.

### Congenital Abnormalities of the Kidney and Urinary Tract

Congenital anomalies of the kidney and the urinary tract (CAKUT), which include a compilation of abnormalities of the upper and lower urinary tracts, represent 30% of prenatal abnormalities.^103^ Within the upper urinary tract, common anomalies include renal changes (dysplasia, agenesis, hypoplasia, ectopia, fusion, duplication, and supernumerary kidneys), ureteral anomalies (ureterocele, vesicoureteral reflux, primary megaureter, ureteropelvic or ureterovesical junction obstruction, and ureteral duplication), posterior urethral valves, and hypospadias.^104^ *FAT4* is a gene implicated in CAKUT and has associations with cryptorchidism and subsequent spermatogenic failure.^105^ Interestingly, whole-exome sequencing (WES) of patients with CAKUT has revealed a range of additional phenotypes outside the urinary system, including facial dysmorphisms, cleft palate, microcephaly, gastrointestinal abnormalities, intellectual disability, hypotonia, and skeletal deformity.^105^

### Myc-Associated Zinc Finger Protein

Myc-associated zinc finger protein (*MAZ*), located on chromosome 16p11.2, is a gene that encodes a C2H2 zinc finger transcription thought to impact WNT signaling.^106,107^ *MAZ* related abnormalities occur in a dosage-sensitive fashion; although it is expressed ubiquitously throughout the body, it has been found to cause genitourinary birth defects even in non-syndromic individuals.^15^ *MAZ* was originally only thought to be a simple housekeeping gene; deletion of *MAZ* resulted in defective development of the genitourinary system in patients exhibiting cryptorchidism, micropenis, and bladder maldevelopment.^15^ Copy number variants of *MAZ* have been associated with issues in other organ systems, and individuals with *MAZ* copy number variants exhibit behavioral abnormalities, cardiac anomalies, gastrointestinal issues, skin and hair changes, ocular problems, and facial dysmorphisms.^9^

### CRK-Like Proto-Oncogene

CRK-like proto-oncogene (*CRKL*) is associated with the DiGeorge/del22q11.2 syndrome and encodes a SH2 and SH3 homology adaptor protein, which is involved in tyrosine kinase signaling pathways.^11^ This gene is expressed ubiquitously throughout the body, but it has been discovered that *CRKL* deletion is responsible for upper urinary tract abnormalities in addition to cryptorchidism and micropenis.^11^ Although a cryptorchid phenotype was observed, spermatogenic failure did not occur, suggesting that *CRKL* had a unique role in fertility and spermatogenesis.^11^ *CRKL* mutations affect other organ systems as well and causes defects, including cardiac defects, craniofacial anomalies, hearing and ocular changes, development impacts, endocrine dysfunction, liver problems, and gastrointestinal dysfunction.^9^

### Congenital Bilateral Absence of the Vas Deferens

Abnormalities in the *CFTR* gene, located on chromosome 7, are a well-documented source of male infertility.^108,109^ The most common mutation within the gene of over 1300 different possible mutations is phenylalanine at position 508, which results in abnormal protein folding and dysfunction of a chloride channel.^110^ Given multiple possible mutations, various phenotypes are possible and include chronic bronchiectasis with recurrent infections and pancreatic insufficiency.^111^ Polymorphisms in addition to gene mutations, such as the 5T allele, also modify protein expression impacting RNA splicing, protein translation, and penetrance.^10^

Infertility in this subset of men is often secondary to obstructive azoospermia owing to congenital bilateral absence of the vas deferens (CBAVD) but may also be because of atrophy and/or absence of other key structures in the reproductive tract, such as the seminal vesicles or epididymis.^112^ Although an overwhelming majority (>97%) of men with cystic fibrosis have CBAVD, a large number of men possessing a *CFTR* mutation have no significant stigmata of the disease.^113^ Given that the vas deferens develops from the mesonephric duct, these men may have other genitourinary changes, including renal agenesis.

A majority of CBAVD cases (80%) are related to *CFTR* mutations; however, the remaining (20%) possess no clear etiology.^114^ WES discovery of the CFTR gene permitted the identification of adhesion G protein-coupled receptor G2 (*ADGRG2*) on the X chromosome at locus p22.13.^115^ *ADGRG2* is part of a family of receptors throughout the body but specifically appears to have a tissue-restricted pattern to the efferent ducts with gene deletion, providing a possible etiology of infertility in these men.^114^

### Other Genes Implicated in Birth Defects

Numerous other genes have been identified and are responsible for both genitourinary birth defects and other systemic conditions, and many of them continue to be investigated. Examples of such genes include mutations of *E2F1*, which is implicated in spermatogenic failure and cryptorchidism, *OTX1*, which is associated with external genitalia birth defects and renal anomalies, as well as *KANK1, KCTD13*, and *SH2B1* (Table 2).^9^

## Other Conditions

### Myotonic Dystrophy

Myotonic dystrophy is an autosomal dominant condition involving a trinucleotide CTG repeat and occurs secondary to an abnormality in 1 of 2 genes, *DMPK* (type 1) or *CNBP* (type 2).^116^ Myotonic dystrophy symptoms may appear early in life or not until later in adulthood and mainly include muscular weakness. Additional problems affecting the individuals with this disease include cardiac abnormalities, endocrinopathies, developmental delay, and cataracts.^117^ Larger CTG expansions may confer more severe phenotypes.^118^ Type 1 has been implicated in infertility and type 2 with hypogonadism. These individuals have testicular atrophy along with hyalinization and atrophy of the seminiferous tubules on histopathologic analysis, which could lead to infertility.^117^

### Noonan Syndrome

This disorder affects many body systems and is associated with infertility. Individuals usually have unusual facial features, short stature, cardiac and renal abnormalities, developmental delay, coagulation disorders, lymphatic malformations, skeletal abnormalities, and genetic predisposition to myeloproliferative disorders.^119^ The majority (approximately 50%) of individuals with Noonan syndrome have a missense mutation in the protein of tyrosine phosphatase non-receptor type 11 (*PTPN11*) gene, whereas up to 30% of them may have no identifiable genetic cause.^120^ From a reproductive perspective, these men present with testicular Leydig cell dysfunction and altered hormonal levels, such as an elevated follicle-stimulating hormone.^121^ Furthermore, these men often have bilateral cryptorchidism, which can lead to spermatogenic dysfunction.^121^

### Spina Bifida

Spina bifida is also known as myelomeningocele, and individuals with spina bifida have incomplete closure of the s pinal cord with variable degrees of severity from spina bifida occulta (small gap in the spine with no entrapment of cerebrospinal fluid or spinal-cord contents) to myelomeningocele, which includes spinal cord exposure in the region of the lumbar spine.^122^ Although these patients do not have testicular dysfunction, depending on the degree of spinal cord involvement and hydrocephalus, they may have sexual dysfunction owing to ejaculatory failure and possible fertility issues.^123^ Therefore, these patients may require electro- or vibratory-stimulated ejaculation or surgical sperm retrieval to obtain sperm for assisted reproduction. In rare cases, there have been reports of spermatogenic deficiencies with an unknown etiology because the testes are generally normal.^123^

### Bladder Exstrophy

Bladder exstrophy occurs in 1/30 000 to 1/50 000 of live births. This rare condition includes incomplete closure of the lower anterior abdominal wall resulting in externalization of the urinary bladder and epispadias secondary to inadequate formation of the urethra.^124^ Although testicular function and spermatogenesis are normal, men with bladder exstrophy often have reduced penile length, which may affect sexual function, and epispadias may create anatomical challenges for natural conception.^125^ There have been some isolated reports of patients with exstrophy with azoospermia.^125^

### Prune Belly Syndrome

Prune belly syndrome includes a triad of cryptorchidism, urinary tract malformations, and reduced abdominal wall musculature. This rare condition is estimated to occur in 1/30 000 to 1/40 000.^126^ Secondary to cryptorchidism, these individuals have spermatogenic dysfunction but may also have ejaculatory dysfunction because of their megalourethra and reduced antegrade ejaculation from bladder neck incompetence.^127^

## Conclusion

Male factor infertility is relatively common, and men with infertility may harbor systemic and genetic diseases. These men warrant a thorough workup and evaluation to assess for additional systemic diseases because they could have a subtle phenotype and/or be asymptomatic. Infertility may be the presenting symptom of the underlying disease, and identification of other medical issues during infertility workup may permit early intervention and limit further disease progression.

## Figures and Tables

**Table 1.** Systemic Diseases and Conditions Associated with Y Chromosome Gene Alterations

Gene	Location	Systemic Disease Association
DBY	Yp; AZFa	Expressed in human serum after ischemic stroke;^26^ expressed in the brain, may be a biomarker for Parkinson’s disease.^27^
USP9Y	Yp; AZFa	Upregulated in heart failure and dilated ischemic cardiomyopathy^28^
UTY	Yp; AZFa	Predisposes men to higher risk of coronary artery disease;^29^ associated with urothelial cancers of the genitourinary tract in animal models^30^
EIF1AY	Yp; AZFb	Expressed in human serum after ischemic stroke;^26^ upregulated in heart failure and dilated ischemic cardiomyopathy^28^
KDM5D	Yp; AZFb	Altered gene expression and epigenetic modifications results in aggressive prostate cancer^31^
RBMY	Yp; AZFb	Linked to male hepatocellular carcinoma^32^
TSPY	Yq	Linked to early- and late-stage gonadoblastoma and germ-cell tumors^21^
SHOX	PAR1	Birth defects: Leri-Weill dyschondrosteosis, Madelung deformity of wrists, short stature^33,34^
VAMP7	PAR2	Birth defects: anomalies of external male genitalia (reduced penile length, hypospadias, and cryptorchidism) and autism spectrum disorder^12^

**Table 2.** Gene Mutations and Copy Number Variants and Their Known Associations with Male Genitourinary Birth Defects

Gene	Location	Function
E2F transcription factor 1 (E2F1)	20q11.22	Transcription factor involved in cell-cycle regulation and apoptosis
Orthodenticle homeobox 1 (OTX1)	2p15	Transcription factor with roles in the vertebrae, brain, and development of sensory organs
Kidney ankyrin repeat-containing protein 1 (KANK1)	9p23	Involved in cytoskeleton formation via actin polymerization
Potassium channel tetramerization domain containing 13 (KCTD13)	16p11.2	Substrate adapter of a E3 ubiquitin protein ligase
SH2B adaptor protein 1 (SH2B1)	16p11.2	Adaptor protein that binds to tyrosine kinases
